# Electronic cigarette devices targeting youth in Korea

**DOI:** 10.18332/tpc/151549

**Published:** 2022-08-04

**Authors:** Sodam Chu, Jaehyung Kong

**Affiliations:** 1 National Tobacco Control Centre, Korea Health Promotion Institute, Seoul, Republic of Korea

**Keywords:** tobacco, e-cigarette, youth, denormalization, health warning

**Dear Editor,**

Tobacco companies have targeted young age groups as growth engines for their future business, and have long invested and made efforts to the cigarette packaging^1,2^. Since consumers are directly exposed to cigarette packs, they themselves have the effect of product advertising and are an important marketing resource to raise awareness of the brand. Tobacco is a ‘badge product’, a way of expressing self-identity in the younger generation, and is a tool to express youth’s individuality and personality^3,4^. WHO Framework Convention on Tobacco Control (WHO FCTC) recommends implementing a health warning with images and texts on cigarette packs, and about 90% of the Parties to the Convention currently implement the measure^5^. Furthermore, 17 countries have introduced and implemented a plain packaging policy that standardizes materials, shapes, and colors so that advertisements and promotional elements cannot be used on cigarette packaging^6^. However, this applies only to cigarettes while packaging regulations for e-cigarettes are scarce. In the meantime, a marketing strategy of ‘advertising through cigarette packaging’ has been equally applied to e-cigarettes. According to recent statistics, the prevalence of current cigarette use among young people (19–29 years old) in South Korea has decreased to 24.8% but the current rate of e-cigarette use is 6.3%, which has been on a steady rise since 2013^7^. The emergence of e-cigarettes has attracted the curiosity of young people^8^ and has set the fashion. The public prefers e-cigarettes to cigarettes in terms of the sophisticated design and technology offered by tobacco companies^9^ and the tobacco companies are devoted to the ‘cigarette packaging strategy’ to stimulate public sentiment and consumption^2^.

As a result of this study, it was found that two major product packaging strategies were used to target the youth. The first is Brand Bundling. This is to put the brand image preferred by young people on e-cigarettes. For example, e-cigarette manufacturers and related industries sell stickers, cases and key rings with an image of a luxurious brand like Tonino Lamborghini and Louis Vuitton to allow people to customize their e-cigarette devices (Figure 1). This is not much different from past cigarette packaging marketing. KT&G, South Korea’s No. 1 tobacco company, once launched a premium cigarette brand with the Toro Emblem, attempting to utilize the charisma of the Lamborghini family, at the top of its product package (Figure 2). It also launched a fashionable and sensuous cigarette package in collaboration with ‘PLAC JEAN’, a popular casual brand among young consumers for its slim-fit jeans, utilizing various patterns used in actual clothing. Through this ‘Brand Bundling’ strategy, tobacco companies meet young people’s desire to customize and own the product to enjoy the intended image and to show off.

**Figure 1:**
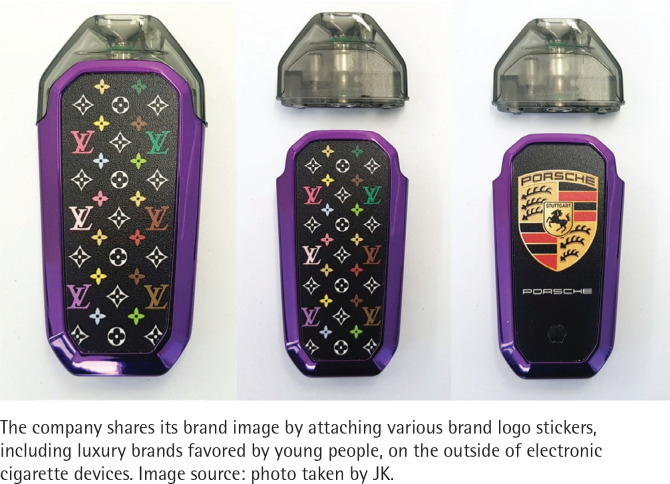
E-cigarette products with major famous brand logo stickers

**Figure 2:**
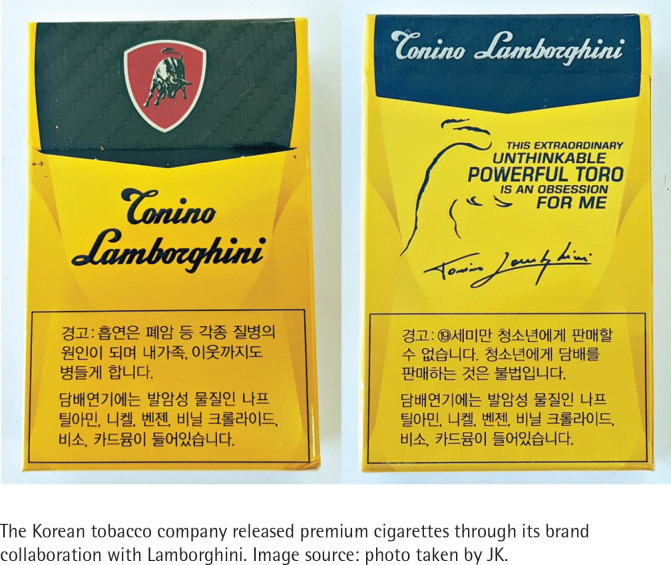
Example of cigarette brand collaboration using Lamborghini logo

The second type is ‘Daily’ strategy to neutralize tobacco denormalization. E-cigarette manufacturers continue to launch different devices in various shapes, not limited to the those similar to existing tobacco products. Devices such as watch-type, car key-type, game console-type, glow-in-the-dark, computer mouse-type and cosmetic container-type are available in the South Korea tobacco market (Figure 3). Common tactic behind these products is that they mimic the products we use in our daily lives. Young adults easily perceive the e-cigarette itself as a ‘toy’ through the appearance and aesthetic elements similar to personal electronic products in daily life, which can serve as an opportunity to promote the use of e-cigarettes^10,11^.

**Figure 3:**
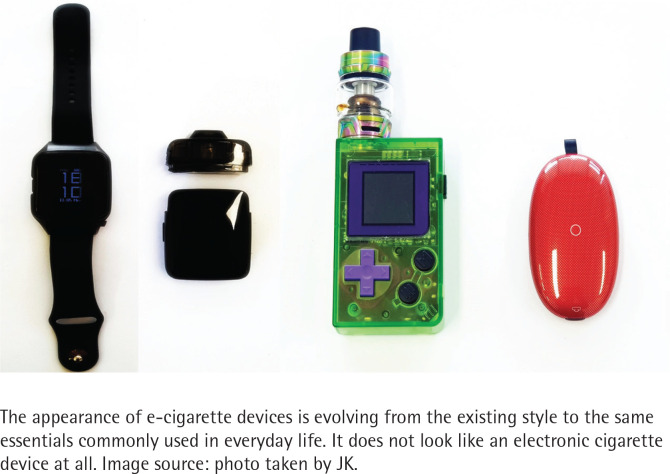
New type electronic cigarette device designed as daily necessities

The marketing strategy of e-cigarette manufacturers and related industries is rapidly evolving. However, tobacco regulations are not evolving in line with the market changes and are not preemptive. In South Korea, e-cigarettes or heated tobacco products are classified as tobacco products by law^12^, and the same regulations as cigarettes are applied. However, e-cigarette devices are classified by law as general consumer products rather than tobacco products, so there is no obligation to print and inscribe health warnings, and they are not subject to tobacco regulations such as taxation and advertising regulations. The appearance of a fancy, sophisticated e-cigarette device is feared to turn into a ‘it-item’, rather than a simple device for tobacco use in the younger age group, as a means to maintain interpersonal relations and a popular cultural item. What is more problematic is that tobacco companies are well aware of the blind spots in the law and are using this to aggressively advertise and promote cigarettes through e-cigarette devices, but they cannot be regulated^13^. Tobacco companies can carry out aggressive advertising and marketing targeting of e-cigarette devices, which are blind spots of the law, so that no regulations can be imposed even though they have the same effect as regular cigarette advertising.

Therefore, e-cigarette devices need to be regulated in accordance with ‘tobacco’. Based on South Korea’s case, WHO FCTC and the Parties to the Convention should strengthen tobacco control policies by reviewing measures such as expanding tobacco definitions, including e-cigarette devices, mandating the attachment of health warnings for e-cigarette devices, taxation and banning advertising and marketing.

## Data Availability

Data sharing is not applicable to this article as no new data were created.

## References

[1] UCSF (1983). The importance of younger adults. Truth Tobacco Industry Documents.

[2] Wakefield M, Morely C, Horan JK, Cummings KM (2002). The cigarette pack as image: new evidence from tobacco industry documents. Tob Control.

[3] DiFranza JR (1995). The effects of tobacco advertising on children. Tobacco and Health.

[4] Gerard H, Karine GM, Juan MR (2008). The plain truth about tobacco packaging. Tob Control.

[5] World Health Organization (2022). 2021 global progress report on implementation of the WHO Framework Convention on Tobacco Control.

[6] World Health Organization (2021). WHO report on the global tobacco epidemic 2021: addressing new and emerging products.

[7] (2020). Korea Centers for Disease Control and Prevention. Korea Health Statistics 2019.

[8] Brown R, Bauld L, de Lacy E (2020). A qualitative study of e-cigarette emergence and the potential for renormalization of smoking in UK youth. Int J Drug Policy.

[9] Okawa S, Tabuchi T, Miyashiro I (2020). Who Uses E-cigarettes and Why? E-cigarette Use among Older Adolescents and Young Adults in Japan: JASTIS Study. J Psychoactive Drugs.

[10] McDonald EA, Ling PM (2015). One of several ‘toys’ for smoking: young adult experiences with electronic cigarettes in New York City. Tob Control.

[11] Keamy-Minor E, McQuoid J, Ling PM (2019). Young adult perceptions of JULL and other pod electronic cigarette devices in California: a qualitative study. BMJ open.

[12] Korea Ministry of Economy and Finance. Tobacco Business Act.

[13] Willett JG, Bennett M, Hair EC (2019). Recognition, use and perceptions of JULL among youth and young adults. Tob Control.

